# Fertility after hysteroscopic management of retained intrauterine bones: a case report

**DOI:** 10.11604/pamj.2025.52.73.49424

**Published:** 2025-10-16

**Authors:** Anny Ngassam, Esther Juliette Ngo Um Meka, Obase Musono Ralph, Diane Estelle Modjo Kamdem, Serge Robert Nyada, Etienne Belinga, Jean Dupont Ngowa Kemfang

**Affiliations:** 1 Centre Hospitalier de Recherche et d´Application en Chirurgie Endoscopique et Reproduction Humaine, Yaoundé, Cameroun,; 2 Département de Gynécologie-Obstétrique, Faculté de Médecine et des Sciences Biomédicales, Université de Yaoundé I, Yaoundé, Cameroun,; 3 Département de Gynécologie-Obstétrique et Santé Maternelle, Faculté de Médecine et des Sciences Pharmaceutiques, Université de Dschang, Dschang, Cameroun

**Keywords:** Fertility, hysteroscopic management, intrauterine fetal bones, case report

## Abstract

Retained intrauterine fetal bone is a rare event that happens after an induced pregnancy termination, usually illegal, especially during the second and third trimesters, due to the incomplete evacuation of fetal tissues. It can cause secondary infertility as bone fragments can work as an intrauterine contraceptive device. We present a case of compromised fertility due to the presence of fetal bony sequestra in the uterine cavity in a 36-year-old female who presented with secondary infertility of eight years duration, following a history of second-trimester voluntary termination of pregnancy. Diagnostic evaluation included ultrasound and hysterosonography. Hysteroscopy confirmed the presence of a fetal bone fragment in the uterine cavity. The patient underwent operative hysteroscopy under loco-regional anesthesia for the removal of the bony sequestra. Pregnancy occurred six months following treatment, and she delivered a term live baby. Retained intrauterine fetal bones should be considered as a possible cause of infertility in women with a history of second-trimester abortion.

## Introduction

Retained intrauterine bones (RIUB) (the bony womb) is a rare but significant cause of uterine factor infertility [1]. The prevalence of infertility caused by RIUB varies by study and has been reported to be 0.15 - 0.28% [2,3]. In Cameroon, a prevalence of 0.7% over a 5-year period has equally been reported [4]. It is usually associated with a past history of mid-trimester termination of pregnancy; however, other rare causes of RIUB include osseous endometrial metaplasia, dystrophic endometrial calcifications, and heteroplasia secondary to hypercalcemia, hyperphosphatemia, and hypervitaminosis D [3,5]. RIUB causes secondary infertility by acting as a foreign body (an intrauterine device), thereby causing a localized inflammation through the release of endometrial prostaglandin F2-α, and thus preventing implantation [6]. Other possible mechanisms include mechanical obstruction of the endometrial cavity or direct embryo toxicity due to osseous particles [3].

Common presenting problems as a result may include inability to conceive (infertility), menstrual irregularities, abnormal uterine bleeding, dysmenorrhea, vaginal discharge, dyspareunia, and chronic pelvic pain [3,6]. On ultrasound, the lesions usually appear as hyperechoic foci of the uterine cavity or an intrauterine foreign body [2,6]. Hysteroscopy is the gold standard, and it is both diagnostic and therapeutic, and fertility is restored in most of these patients following hysteroscopic removal [5]. We report a case of secondary infertility caused by retained intrauterine fetal bones.

## Patient and observation

**Patient information:** we present the case of a 36-year-old married woman, G1P0010, who presented at the outpatient consultation in our hospital with an inability to conceive for the past 8 years, associated with hypomenorrhea. Her past history was relevant for voluntary termination of a 16-week pregnancy by dilatation and curettage 10 years earlier. She has a regular 28 - 30-day menstrual cycle with menses initially lasting for 3 - 5 days, except for the past 8 years when menses progressively became short and scanty. Her partner is a 45-year-old male, and they live together. He has a remarkable history of varicocelectomy and abnormal semen analysis.

**Timeline of the current episode:** ten years prior to presentation, the patient had a voluntary termination of a 16-week gestation at a peripheral health facility. The couple has been married for eight years and presents with primary infertility. They have consulted several healthcare centers without achieving a favorable outcome. Initial investigations were limited to infectious screenings and low-quality pelvic ultrasounds for the female partner. The male partner's semen analysis revealed severe abnormalities. The couple received empirical treatment with antibiotics, undocumented antioxidants, and ovulation inducers, but no significant improvement was observed. Due to the persistence of infertility, the male partner was referred to a urologist. A testicular ultrasound revealed a varicocele, which was managed surgically. A slight improvement in semen parameters was noted postoperatively. Despite this favorable andrological development, no spontaneous conception occurred. The couple was therefore referred to our unit for specialized management of their infertility.

**Clinical findings:** her general physical examination was unremarkable. The cervix was macroscopically normal on speculum examination, and the uterus was of normal size, with free adnexa on bimanual vaginal examination.

**Figure 1:**
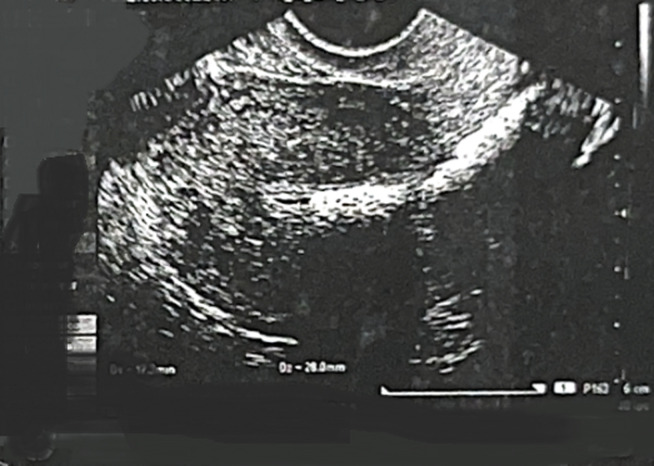
trans-vaginal ultrasound scan showing dense linear hyperechoic substances in the endometrium with acoustic shadowing

**Figure 2:**
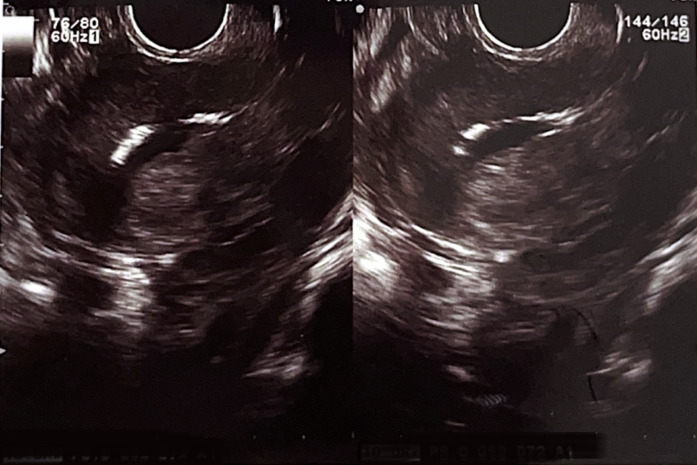
saline infusion sonography showing dense linear hyperechoic substances in the endometrium with acoustic shadowing

**Diagnostic assessment:** among her initial fertility workups was a transvaginal ultrasound and saline infusion sonography, which revealed dense linear echogenic structures in the endometrium with acoustic shadowing (Figure 1, Figure 2).

**Figure 3:**
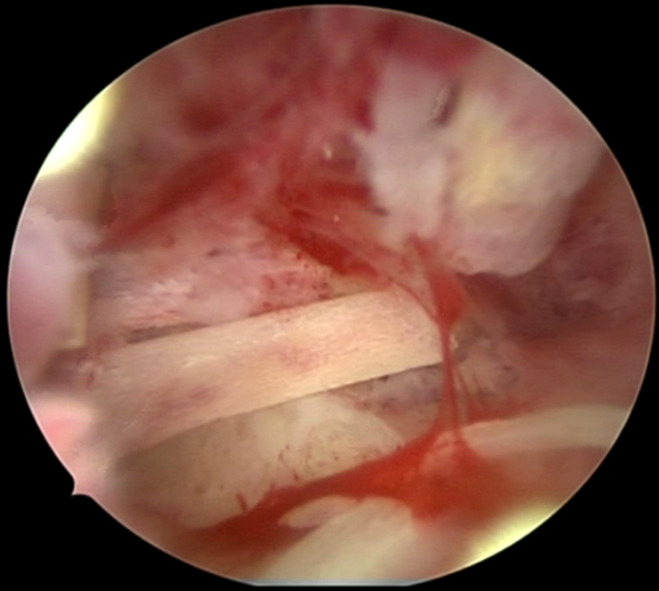
hysteroscopic view of bone-like foreign bodies in the endometrium

**Figure 4:**
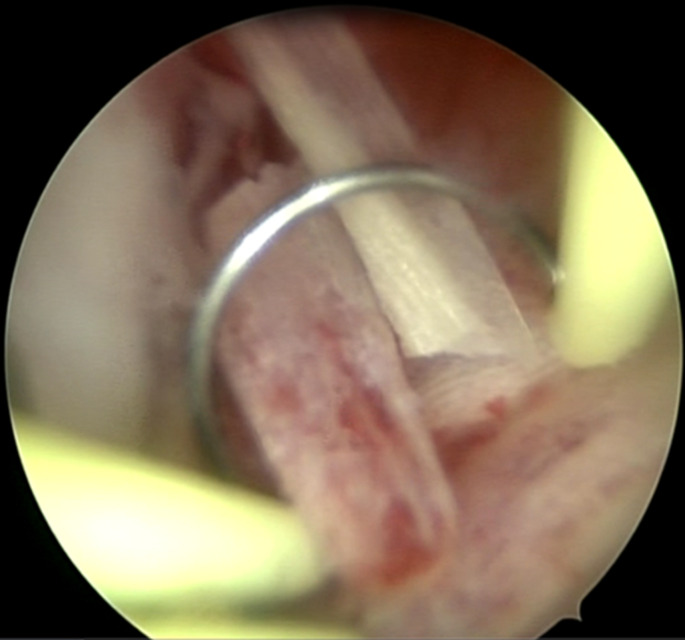
hysteroscopic removal of bony spicules in the endometrium using a diathermy loop resectoscope

**Therapeutic interventions:** she subsequently underwent a diagnostic hysteroscopy with the use of a Bettochi (a continuous flow panoramic rigid hysteroscope, 26 cm in length, 2.9 mm of outer diameter sheath and 30° fiberoptic lens Endoscope). Therapeutic hysteroscopy was done using a resectoscope with a diathermy loop. The loop was passively used to withdraw bone fragments without energy activation. Distension of the uterine cavity was achieved with normal saline. The intraoperative findings were remarkable for bone-like foreign bodies in the endometrium (Figure 3). A diagnosis of intrauterine fetal bone retention was made, and the residual bony spicules were removed with the aid of a diathermy loop resectoscope (Figure 4). An endometrial biopsy was equally performed, and the histopathological evaluation of the specimens revealed an inflamed endometrium with bony remnants characterized by mature degenerated osteocytes, bone tissue, and calcification.

**Follow-up and outcome of interventions:** the patient was administered antibiotics for 21 days and estrogen-progesterone pills for 3 months, and her menstrual cycle was normalized. She later conceived through an in vitro fertilization, and delivered a live term male baby through a lower uterine segment cesarean section.

**Patient perspective:** *It was initially difficult for me to believe the fact that there could be bones in my womb, but my doubts were clarified after the operation. The diagnostic and treatment processes were a little stressful, but I appreciated the clear explanations and reassurance provided by the medical team. Looking back, I realize the dangers and complications of criminal abortion and I hope sharing my story can encourage other patients with similar conditions*.

**Informed consent:** a written informed consent was obtained from the patient for publication of this case report, and a copy is available for review by the editor of this journal.

## Discussion

Retained intrauterine fetal bone is a rare event that happens after an induced pregnancy termination, especially during the second and third trimesters, due to the incomplete evacuation of fetal tissues [5]. The finding of bone within the uterus or endometrium has been described as ossification of the endometrium, osseous metaplasia of endometrium, ectopic intrauterine bone, and heterotopic intrauterine bone [7].

Retained intrauterine bones (RIUB) (the bony womb) is a rare but significant cause of uterine factor infertility [1]. The prevalence of infertility caused by RIUB varies by study and has been reported to be 0.15 - 0.28% [2,3]. In Cameroon, a prevalence of 0.7% over a 5-year period has also been reported [4]. Termination of pregnancies in Cameroon is illegal, and such operations, therefore, are more likely to be performed by inexperienced practitioners and those with no medical qualifications, thus carrying a greater risk of being incomplete.

These retained bones may act as ‘uterine synechia´ or intrauterine copper device, thereby causing a localized inflammation through the release of endometrial prostaglandin F2-α, and thus preventing implantation [6]. Other possible mechanisms include mechanical obstruction of the endometrial cavity or direct embryo toxicity due to osseous particles [3]. The risk of infertility depends on the location of the retained pieces of bone, whether it is embedded in the myometrium or in the endometrial cavity. Some evidence exists that the presence of an intramural bony fragment per se does not seem to compromise fertility if it is completely embedded.

Infertility can significantly negatively impact a woman´s quality of life, leading to substantial emotional distress, relationship difficulties, social isolation, low self-esteem, and feelings of loss due to the inability to conceive a child. This may further affect various aspects of life, including work, family, and personal well-being, particularly in women who experience greater societal pressure to become mothers. Although such features were not explored in our case, one could not invariably exclude them, especially in a woman who presented with a desire to conceive for over 8 years.

Following hysteroscopic removal of the bony spicules, she was placed on estrogens and progestatives for three months to enhance endometrial regeneration. This treatment equally resulted in a normal regular menstrual cycle. According to one systematic review by Khan *et al*. in 2016 on bone in the endometrium, fertility is usually improved following treatment, and the pregnancy rate is around 76.6%, and the majority (74/90 (82.2%)) of these women conceived spontaneously, while 10/90 (11.1%) conceived through assisted methods of reproduction [8]. They also found that most of these pregnancies were achieved within 6 months following treatment [8]. The findings in our case corroborate findings in the literature. In our case, fertility was achieved three months following treatment through in vitro fertilization. This was so because of an associated male-factor infertility.

A simple ultrasound-based approach to investigate the infertile patient can be used effectively as an initial examination modality during the couple´s workup. It is the best imaging technique that can readily visualize retained bone in the endometrium. Fetal endochondral ossification usually occurs from 12 weeks of gestation. If there is termination of pregnancy or spontaneous abortion after this period, retained bone fragments will be displayed as bright echogenic areas with posterior shadowing [9]. In our case, the ultrasound revealed multiple dense linear echogenic structures in the endometrium with acoustic shadowing, which were consistent with the hysteroscopic findings. A regular myometrial-endometrial interface and homogenous endometrial structure on transvaginal sonography, congruent with the phase of the menstrual cycle, indicate a normal endometrium and preclude the need for diagnostic hysteroscopy [9].

Other possible differential diagnoses on sonography could be uterine synechiae or osseous endometrial metaplasia. Uterine synechiae, however, appear as thick, echogenic bands connecting the uterine walls, while osseous metaplasia is visualized as hyperechoic linear or irregular areas within the endometrium [10]. Hysteroscopy, however, remains the gold standard as it has both accurate diagnostic and therapeutic values. It allows for direct visualization of the endometrial cavity, and retained intrauterine fetal bones are viewed as bony spicules in the endometrium [8]. Other diagnostic modalities reported in the literature include hysterosalpingography and hysterosonography [8]. In terms of pathology, minimal adjacent endometrial reaction with endochondral ossification may help to distinguish osseous metaplasia from retained fetal osteoblastic tissue after an abortion [6].

## Conclusion

In women presenting with difficulties conceiving with a history of second-trimester abortion, retained intrauterine fetal bones should be considered as one of the causes. Routine transvaginal ultrasound evaluation forms an excellent diagnostic tool for such patients. Hysteroscopic removal remains the gold standard for management of this condition, and fertility is generally improved if managed accordingly.
